# Key informant interview on antimicrobial resistance (AMR) in some countries in the western pacific region

**DOI:** 10.1186/1744-8603-9-34

**Published:** 2013-07-26

**Authors:** Yuri Lee, Mami Wakabayashi

**Affiliations:** 1 Health Services Development Unit, World Health Organization, Western Pacific Regional Office, United Nations Avenue, P.O. Box 2932, Manila, 1000, Philippines; 2 Institute of Health Services Research, Yonsei University, Seoul, South Korea

**Keywords:** Antimicrobial resistance (AMR), Key informant interview (KII), Western pacific region, World health day, World health organization (WHO)

## Abstract

**Background:**

The World Health Organization (WHO) selected antimicrobial resistance (AMR) as the theme for World Health Day 2011. The slogan was “Combat Drug Resistance – No action today, no cure tomorrow” A six-point policy package was launched as a core product for World Health Day. It aimed to stimulate extensive and coherent action to overcome the many challenges presented by antimicrobial resistance.

**Methods:**

As a preparation for World Health Day, interviews were conducted with a series of key informants, mainly senior government staff, to assess their awareness of the topic and the interventions proposed in the policy package. Since the key informant interview methodology was used with a small number of interviewees, it may be difficult to demonstrate the validity of the findings.

**Results:**

Key informants from twelve out of fifteen countries responded, which included Fiji (n = 5), Kiribati (n = 1), Lao PDR (n = 2), Malaysia (n = 6), Micronesia (n = 3), Mongolia (n = 5), the Philippines (n = 5), Vietnam (n = 6), Vanuatu (n = 1), Solomon Islands (n = 3), Cambodia (n = 5) and Brunei (n = 1). There was a total of forty-three respondents (n = 43). AMR was widely recognized as a problem. Lack of a coherent, comprehensive and national plan or strategy was noted. Surveillance was often seen as weak and fragmented even where presented. Laboratory capacity was felt to be insufficient across all countries interviewed. The majority of respondents stressed the need for national and local plans to combat AMR including reliable estimates of the financial cost of combating and managing AMR, the need for legislation to control inappropriate use of antimicrobials in food animals and more serious efforts to promote Standard Treatment Guidelines (STGs) and Rational Prescription. Also, importance was highlighted of the need to include infection prevention and control (IPC) as a part of accreditation and registration of health institutions and programs to promote IPC to the general population.

**Conclusion:**

A coalition of interested parties at the local, national and international levels need to generate and sustain the political will to organize a more comprehensive, sustainable, and coherent approach to AMR.

## Background

Among the most important medicines ever discovered, antimicrobial agents have saved millions of lives and improved the outcomes for countless patients since these drugs were introduced in the early 1930s [1]. However, the emergence and spread of resistance in multiple microorganisms have rendered the management of many infectious diseases more difficult [2].

Antimicrobial resistance (AMR) is defined as the resistance of a microorganism to an antimicrobial medicine to which it was previously sensitive [3,4]. Resistant organisms including bacteria, viruses and some parasites are able to withstand the attack by antimicrobial medicines, such as antibiotics, antivirals, and anti-malarials, so that standard treatments become ineffective and infections persist and may spread to others [3]. The development of resistance to antimicrobials may be inevitable due to natural selection and evolutionary pressure [5-8]. However, there is little doubt that the overuse and misuse of antimicrobials have hastened the development of AMR [3,9,10].

The world has recognized for many years that antimicrobial resistance is a major problem and that comprehensive and coordinated action is desirable [11]. In 2001, the World Health Organization (WHO) published the *WHO GLOBAL STRATEGY FOR CONTAINMENT OF ANTIMICROBIAL RESISTANCE* along with a series of recommendations aimed at enabling countries to define and implement national policies in response to antimicrobial resistance [4].

In 2002, the Regional Committee for the Western Pacific passed a resolution on antimicrobial resistance that recognized the WHO Global Strategy for Containment of Antimicrobial Resistance [12]. The resolution urged the Member States to develop and implement multisectoral strategies to contain antimicrobial resistance, make antimicrobials available only on prescription, and promote and ensure the rational use of drugs [11,13].

The World Health Assembly resolutions related to AMR in 1998, 2005 and 2009 and from various WHO Regional Committee resolutions have been passed, but the consensus is that national and global responses have been inadequate [12]. Strategies for containment, with a few exceptions, have not been widely or effectively implemented. While the actions needed are clear, a commitment to be accountable and implement these strategies has been lacking. The World Health Day, held annually on April 7, is the flagship advocacy event of the, of which theme in 2011 was antimicrobial resistance [14]. The intention for this theme was to partially alleviate the lack of sufficient attention to AMR. A six-point policy package launched on the World Health Day 2011 was aimed at engaging all of WHO’s Member States and the global health community to foster action for change [11].

The six-point policy package was proposed as a core product for World Health Day. It was developed from the WHO Global Strategy for Containment of AMR [15]. The package was distilled from the existing strategy, but aimed to be more direct, more coherent and easier to understand. The package was built around identified problems and proposes actions to address them. It also recognized that the action stimulated by previous WHO resolutions and strategies had been inadequate and aimed to stimulate extensive and coherent action to overcome its present challenges [3].

The six items in the six-point policy package were: (1) to commit to a comprehensive, financed national plan with accountability and civil society engagement, (2) to strengthen surveillance and laboratory capacity, (3) to ensure medicines of good quality and of regular supply, (4) to regulate and promote the rational use of medicines including those in animal husbandry and ensure proper patient care, (5) to enhance infection prevention and control, and (6) to foster innovations and research and development of new tools [12].

The Western Pacific Regional Office (WPRO) of WHO, as a part of its preparation for World Health Day, commissioned key informant interviews with key policy makers in the area of AMR in individual member states. The purpose of the interview was for the key informants to assess the relevance and suitability of the proposed policy package. It also provided an opportunity to see if senior decision makers had knowledge about whether or not the proposed key aspects of the AMR policy package were in place and to sensitize them on the need of the AMR policy package to guide actions against AMR at the country level.

## Methods

The Health Services Development unit developed an instrument for use in conducting key informant interviews. The questionnaire for structure interview was commented upon and refined by members of the AMR Working Group of the WPRO.

Key informant interviews (KII) involve interviewing a select group of individuals who are likely to provide needed information, ideas, and insights on a particular subject. Because information comes directly from knowledgeable people, KII often provide data and insight that cannot be obtained by other methods. But, because KII provide only a very limited basis for quantification, they are rarely appropriate when quantitative data are needed. Moreover, having a small sample pool may prove difficult to demonstrate the validity of the findings [16].

The instrument followed the format of the six-point policy package and consisted of a quantitative and a qualitative part. Each of the six-points had 5–6 closed-ended questions which required a “Yes”, “No”, or “Do Not Know (DNK)” answer. These were then linked to an open-ended question on the same topic that was used to explore the respondent’s answer further. The questionnaire was an applied mixture of both self-reporting and face-to-face interviews conducted by the Health Services Development unit of WPRO, WHO.

The interviewees were identified by the WHO country offices in their respective regions in cooperation with the focal point in the Ministry of Health (MoH). The interviewees were typically a senior researcher from a local research institute or an official from the MoH. The task involved identifying the officials from the MoH in different departments. Given the time constraints in accessing senior policy makers, the focal point persons from the MoH and WHO were asked to help make arrangements for the interviews. Due to the time limits and budget constraints, it was not possible to carry out interviews with the private sector and professional associations.

The questionnaire could be completed by the key informant independently or through a face-to-face interview. An Interview took about 30 minutes. Key informants felt comfortable with the “Yes”, “No”, and “DNK” questions, but for the more detailed comment questions, some felt unease as it required more time and a more thorough discussion. The interviews were conducted only in the Member States of the Western Pacific Region where the WHO had an office, which meant that the majority of the responses gathered were from low and middle-income countries. The plan was to have three to five interviews per country, with five from the larger countries and three from the smaller.

## Results and discussion

Interviewees from twelve out of fifteen countries responded, which included Fiji (n = 5), Kiribati (n = 1), Lao PDR (n = 2), Malaysia (n = 6), Micronesia (n = 3), Mongolia (n = 5), the Philippines (n = 5), Vietnam (n = 6), Vanuatu (n = 1), Solomon Islands (n = 3), Cambodia (n = 5), and Brunei (n = 1). The total number of respondents was forty-three (n = 43). Respondents identified themselves from a wide variety of professions including pharmacists, medical doctors, physicians, pathologists, medical microbiologists, bacteriologists, public health specialists, health administrators, family medical consultants, laboratory scientists and epidemiologists.

Because small samples were chosen and random sampling methods were not used, it would not be safe to make generalizations about the results. However, the KII provides valuable information for programs, particularly when following the general process and steps. In this study, it first identified stakeholders and what information is needed and from whom. Second, developing an interview protocol, which is the rule that guides the administration and implementation of the interview, was done. Third, interviews with stakeholders were set up after explaining the purpose of the interview, why he/she has been chosen, the expected duration of the interview, while an informed consent was asked of the interviewee.

### Key informants results

The answers to the closed-end questions are presented in detail in Table 1. It is emphasized that the sampling method and sample size did not lead to numbers that are considered statistically significant. However, the data do provide a general view of the opinions of decision makers about the questions which is of value.

**Table 1:** Quantitative data of responses from key informant consultation on AMR

**Question**	**Yes**	**No**	**DNK**
**1. Commit to a comprehensive, financed national plan with accountability and civil society engagement**			
1a. Does your country have a comprehensive national plan to combat or manage the problem of antimicrobial resistance?	4	28	9
	(9.8)	(68.3)	(22.0)
1b. Are local or institutional plans in place for combating or managing antimicrobial resistance even if no national plan?	25	10	5
	(62.5)	(25.0)	(12.5)
1c. Is there a reliable estimate of what the financial cost of combating or managing antimicrobial resistance would be in your country?	4	21	16
	(9.8)	(51.2)	(21.4)
1d. Is it clear to you who (person or body) is responsible for combating or managing antimicrobial resistance in your country?	17	16	9
	(40.5)	(38.1)	(21.4)
1e. Are partners other than the government involved in combating or managing antimicrobial resistance in your country?	19	15	6
	(47.5)	(37.5)	(11.2)
**2. Strengthen surveillance and laboratory capacity**			
2a. Is there a comprehensive AMR surveillance system in your country?	12	23	7
	(28.6)	(54.8)	(16.7)
2b. Are there surveillance systems for AMR in specific organisms such as HIV, malaria, TB, or influenza in your country?	27	5	10
	(64.3)	(11.9)	(23.5)
2c. Is it clear to you who is responsible for AMR surveillance in your country?	21	13	8
	(50.0)	(31.0)	(19.0)
2d. Is there sufficient laboratory capacity in your country to monitory AMR?	13	23	9
	(28.9)	(51.1)	(23.8)
2e. Do you know who is responsible for the laboratory monitoring of AMR?	20	8	12
	(50.0)	(20.0)	(30.0)
2 f. Is there a system of monitoring or surveillance for drug consumption in your country?	17	8	12
	(45.9)	(20.0)	(19.4)
**3. Ensure medicines of good quality and regular supply**			
3a. Does drug quality in your country meet international standards?	27	7	8
	(64.3)	(16.7)	(19.0)
3b. Is there a mechanism to halt or control the sale of counterfeit and substandard medicines?	30	7	5
	(71.4)	(16.7)	(6.5)
3c. Is there a reliable supply or essential medicines to treat infections?	33	3	6
	(78.6)	(7.1)	(14.3)
3d. Is the essential medicine list harmonized or consistent with standard treatment guidelines?	33	2	7
	(76.6)	(4.6)	(16.7)
3e. Is there a method for ensuring that expired or improperly stored drugs are not used?	29	4	9
	(69.0)	(9.5)	(21.4)
**4. Regulate and promote rational use of medicines, including in animal husbandry, and ensure proper patient care**			
4a. Are standard treatment guidelines and continuing education used as part of health provider registration accreditation?	20	14	9
	(46.5)	(32.6)	(20.9)
4b. Are antimicrobials available only by prescription from a trained health worker?	23	18	2
	(53.5)	(41.9)	(4.7)
4c. Is there a method to ban non-recommended monotherapy with key antimicrobials, such as anti-TB, anti-HIV, and anti-malarial drugs?	21	10	12
	(48.8)	(23.3)	(27.9)
4d. Is there legislation to control the inappropriate use of antimicrobials in food animals?	3	20	20
	(7.0)	(46.5)	(46.5)
4e. Is there any public education on appropriate use of antimicrobials?	15	24	4
	(34.9)	(55.8)	(9.3)
**5. Enhance infection prevention and control**			
5a. Do standards for infection prevention and control (IPC) for health care institutions exist?	32	5	4
	(78.0)	(12.2)	(9.8)
5b. Is adequate IPC part of health institution accreditation or registration in your country?	13	22	6
	(31.7)	(53.7)	(14.6)
5c. Is there a continuing education programme to promote IPC among health workers?	25	9	7
	(61.0)	(22.0)	(17.1)
5d. Is there a programme to promote IPC among the general population?	12	23	6
	(29.3)	(56.1)	(14.6)
5e. Do you know who is responsible for IPC in your country?	31	6	4
	(75.6)	(14.6)	(9.8)
**6. Foster innovations and research and development of new tools**			
6a. Is operational research being done to improve the use of existing antimicrobials?	16	14	12
	(38.1)	(33.3)	(28.6)
6b. Is research being done to improve the use of existing diagnostic methods for AMR?	13	17	12
	(31.0)	(40.5)	(28.6)
6c. Is basic research being done to develop new antimicrobials in your country?	4	27	11
	(9.5)	(64.3)	(26.2)
6d. Is basic research being done to develop new diagnostics in your country?	9	19	14
	(21.4)	(45.2)	(33.3)
6e. Is there a process to assess whether new antimicrobials and diagnostic should be introduced?	17	14	11
	(40.5)	(33.3)	(26.2)

Number (%).

The questions focused on the national planning processes indicate that most informants were not aware of a comprehensive national plan and that there was not an estimate available as to the costs of combating AMR. Also, only 40 % of the respondents answered they were aware of which individual or organizational body was responsible for AMR in their country.

The second set of questions revealed a lack of confidence in the existence of comprehensive surveillance systems for AMR, although there was a better response in regards to some disease specific systems being in place. Only 50 % thought they were aware of who is responsible for AMR surveillance while only 28 % felt that laboratory capacity was adequate for AMR surveillance.

A majority of the respondents answered positively to the third set of questions aimed at assessing the ability to ensure medicines of good quality and regular supply. The fourth set of questions dealt with the rational use of medicines to which the informants were less positive. It is of particular interest that only 7 % of respondents felt there was sufficient legislation to control the use of antibiotics in animal feed.

The responses to fifth set of questions dealt with infection prevention and control (IPC) seemed to indicate that there was considerable activity in institutions although IPC was not a part of the health institution accreditation. Informants also felt there was not an adequate program to promote IPC in the general population. The final set of questions dealt with fostering innovation and research. About forty per cent of respondents felt that operational research was being conducted relevant to AMR and that a process existed for the assessment of new antimicrobials and diagnostics. Not surprising for low and middle income countries, the informants had a relatively low-awareness of basic research in respect to AMR.

### Summary of open questions

The first item in the six-point policy package concerns the commitment to a comprehensive, financed national plan with accountability and civil society engagement. The key informants agreed that a comprehensive national plan for AMR is needed and there is a need for developing plans to strengthen existing related activities in order to combat AMR. One respondent was not sure about the feasibility of such plan, perhaps discouraged by the existing fragmentation. The majority said that the local AMR plans should be developed, although one informant felt that given the national guidelines are present, there is no need for local plans. Another informant said that a local plan should be demanded by the national plan. The majority responded that costing is definitely needed and useful. The majority of the sample felt that the MoH or its equivalent within the government should be responsible for AMR, although there is recognition that there are many other stakeholders concerned.

The second item is about strengthening surveillance and laboratory capacity. Most respondents said a comprehensive AMR surveillance system needs to be established and there is a need to develop an institution or department for the improvement of technical capacity for AMR surveillance. Some specific diseases, such as TB, HIV, Influenza, MRSA, VRE, and gonococcus, are established as reportable diseases and have ongoing surveillance. Some of the respondents mentioned surveillance should be expanded to other diseases and be better linked with each other. Many different organizations and departments play a role in surveillance, but some countries have no specific person taking responsibility. In most countries the National Laboratory coordinator in the MoH is responsible for AMR laboratory capacity. However, improving skills and equipment, training, budget, human resources and capacity of all related resources and facilities as well as premises are needed. Moreover some countries have no database for regulation at the national level, but many countries prepare annual reports published by the public sector.

The third item is about ensuring medicines of good quality and regular supply. Many respondents said the national standards on prescribing medications exist, but related law and standards enforcement and their implementation are inadequate. Lack of laboratory capacity, illegal drug use and supply, and procurement systems that fail to comply with standard procedures contribute to AMR. While control measures by regulations and enforcement by the government officials exist for counterfeit and substandard medicines, the implementation of these control measures is imperfect. National drug committees such as the drug and therapeutic committees and disease program managers in the MoH should regularly review essential drug lists (EDLs) and standard treatment guidelines (STGs), but these mechanisms are often not functioning well at the moment. With regard to expired drugs, the majority of the countries’ respondents felt expired drugs are being destroyed appropriately by legal regulations or protocols.

The fourth item concerns the regulation and promotion of the rational use of medicines, including in the area of animal husbandry, and ensuring proper patient care. STGs related to combating AMR are not a part of institutional accreditation or health worker registration for the most part. Most informants agreed that policy development is needed in these areas. Many of the countries have laws for the accessibility of antimicrobials only by prescription, but are not enforced properly, although many informants felt this law was necessary, enforcing this law has been difficult. Most of the countries also do not have legislation to control the inappropriate use of antimicrobials in food animals. The general response from some countries is that this control is partially met by the Ministry of Agriculture (MoA), however key informants from the health sector seemed to have little detail or knowledge in this area. With regard to the general public, key informants believed the public had little knowledge of AMR and had very little access to public education of AMR. They believe public education on AMR through all appropriate mediums is needed.

The fifth item in the AMR policy package is enhancing infection prevention and control (IPC). Most countries have departments in charge of IPC. Some countries, namely Malaysia and the Philippines, have made IPC a part of their institutional accreditation system. While most of the other countries do not have such a system, there is a consensus for the need for its development. Some countries have a system for continuing education for health workers, while others do not. All informants agreed to its need. Most countries do not have IPC programs for the general public, although educational programs on hand-washing are carried out during outbreaks. There seems to be less incentive to continue such programs indefinitely, which would be needed for ongoing issues such as combating AMR. Some countries have specific departments for IPC, while others do not.

The sixth item is on fostering innovations and research as well as the development of new tools. Most of the country informants felt the need for operational research in the use of antimicrobials and diagnostics. Most informants felt that there was not an adequate process to assess whether new antimicrobials and diagnostics should be introduced. The informants felt that basic research to develop new diagnostics and antimicrobials is needed, although those of many countries acknowledged a limited capacity to do such research.

The key informants seemed to uniformly feel that AMR is an important issue and there was a need to develop information systems and infection control policy. AMR was identified as a major challenge to the respective MoH and it was acknowledged that they were not yet operating properly to meet this challenge. The informants identified the need to understand the roles and responsibilities of various parties and to develop sufficient knowledge and strong systems. Many acknowledged the need to make a comprehensive management team and task force responsible for AMR. They also expressed the need to develop a stronger political will to confront the issues.

To the question on what should be expected from the WHO (or other international organizations), the responses included: 1. technical support (training, improvement of the legal environment, human and laboratory capacity, behavior change and advocacy); 2. facilitation of information sharing; 3. advocacy on the international level; 4. capacity building and coordinating in planning country wide training or education programs; and 5. developing instruments, guidelines and policy.

## Key findings and conclusions

The key informants recognized AMR as a problem and that the response to date has been inadequate. They felt that there has been a lack of coherent, comprehensive national planning and strategic processes. Where there has been a response to AMR, it has often been fragmented. AMR surveillance is often shown to be weak, and even where surveillance is stronger, it is often disease-specific and fragmented. Laboratory capacity was felt to be insufficient in all countries interviewed. The majority of respondents stressed the need for national and local plans to combat AMR including reliable estimates of the financial cost to combat and manage AMR. AMR surveillance in the Region will be strengthened by increasing the number of monitoring laboratories in different locations. They also expressed a need for legislation to control the inappropriate use of antimicrobials in food animals, although it was also recognized that the political will needed to be generated for this step to occur.

More serious efforts to promote the STGs and rational prescription were also deemed desirable. The STGs exists in most settings but lacks a strong connection between the STGs and accreditation or licensing. Antibiotics being available without a prescription from a medical practitioner were a problem in most of the countries surveyed. Infection prevention and control activities exist, but many were related to outbreaks. It calls for a movement move to make these activities a part of the daily routine, as AMR is a problem that will continue indefinitely. The strengthening of the capacity and capability of drug regulatory authorities through direct technical support to selected Member States and sharing information through the network and will continue to be an area of priority.

AMR requires a multi-faceted approach that will be sustainable. It is not a problem that can be confronted, defeated and forgotten. It requires action from a wide variety of players from multiple sectors in society. These key informant interviews show that the response to AMR to date is felt to have been inadequate in most countries.

However, this study also indicates that these key informants are aware of most of the related issues and that they have a willingness to tackle them. They accept as clear and relevant the six-point policy package being promoted by the WHO as clear and relevant. However, there is some skepticism as to whether the necessary political commitment on the part of both national and international decision makers needs to be present to actually implement the package. A coalition of interested parties at the local, national and international levels need to generate and sustain the political will to organize and sustain a more comprehensive and coherent approach to AMR.

It is troubling that AMR has been recognized as an important issue for decades [17,18], and that actions that are effective in blunting its impact and slowing its spread are known, but concerted and effective implementation of these actions is still sub-optimal. It remains to be seen whether this situation is changing. Time is not on our side on this issue.

## Key messages

•Antimicrobial Resistance (AMR) is widely recognized as a problem and the need for a comprehensive national plan or strategy was noted.

•Surveillance is often seen as weak and fragmented even where present, and laboratory capacity is felt to be insufficient almost everywhere.

•The majority of respondents stressed the need for national and local plans to combat AMR including reliable estimates of the financial cost of combating and managing AMR, the need for legislation to control inappropriate use of antimicrobials in food animals and more serious efforts to promote the Standard Treatment Guidelines (STGs) and Rational Prescription.

•Also, the importance is highlighted of the need to include infection prevention and control (IPC) as a part of accreditation and registration of health institutions and programs to promote IPC to the general population.

## Competing interests

Both authors declare having no competing interests.

## Authors’ contributions

YL carried out the data analysis and summarization and drafted the manuscript. Both authors read and approved the final manuscript.
